# Publisher Correction: 3D test sample for the calibration and quality control of stimulated emission depletion (STED) and confocal microscopes

**DOI:** 10.1038/s42003-021-02515-1

**Published:** 2021-08-12

**Authors:** Ernest B. van der Wee, Jantina Fokkema, Chris L. Kennedy, Marc del Pozo, D. A. Matthijs de Winter, Peter N. A. Speets, Hans C. Gerritsen, Alfons van Blaaderen

**Affiliations:** 1 Soft Condensed Matter and Molecular Biophysics, Debye Institute for Nanomaterials Science, Utrecht University, Utrecht, the Netherlands; 2 Present Address: Department of Imaging Physics, Delft University of Technology, Delft, the Netherlands; 3 Present Address: Stimuli-responsive Functional Materials and Devices, Department of Chemical Engineering, Eindhoven University of Technology, Eindhoven, the Netherlands; 4 Present Address: Environmental Hydrogeology, Department of Earth Sciences, Utrecht University, Utrecht, the Netherlands

**Keywords:** Super-resolution microscopy, Confocal microscopy

Correction to: *Communications Biology* 10.1038/s42003-021-02432-3, published online 23 July 2021

In the original PDF version of this Article, the image for Fig. 2 was inadvertently replaced with the image from Fig. 3. The online version of the Article was not affected. The correct version of Fig. 2 is  which replaces the previous incorrect version .

This error has now been corrected in the PDF version of the Article.

